# Autologous stem cell transplant for severe, progressive juvenile systemic sclerosis

**DOI:** 10.1093/stcltm/szag005

**Published:** 2026-03-23

**Authors:** Shaikha Alqahtani, Fabiana Cacace, Andrea Bauchat, Brittany Paige DePriest, Timothy Alan Driscoll, Carmem Bonfim, Joanne Kurtzberg, Nicole Larrier, Jeffery Dvergsten, Kris Michael Mahadeo

**Affiliations:** Pediatric Transplant and Cellular Therapy, Duke University, Durham, NC 27705, United States; Pediatric Transplant and Cellular Therapy, Duke University, Durham, NC 27705, United States; Hematopoietic Cell Transplantation and Cellular Therapies Unit, AORN Santobono-Pausilipon, Naples, Italy; Pediatric Transplant and Cellular Therapy, Duke University, Durham, NC 27705, United States; Pediatric Transplant and Cellular Therapy, Duke University, Durham, NC 27705, United States; Pediatric Transplant and Cellular Therapy, Duke University, Durham, NC 27705, United States; Pediatric Transplant and Cellular Therapy, Duke University, Durham, NC 27705, United States; Pediatric Transplant and Cellular Therapy, Duke University, Durham, NC 27705, United States; Department of Radiation Oncology, Duke University, Durham, NC 27703, United States; Department of Pediatric Rheumatology, Duke University, Durham, NC 27703, United States; Pediatric Transplant and Cellular Therapy, Duke University, Durham, NC 27705, United States

**Keywords:** juvenile systemic sclerosis, autologous stem cell transplant, refractory autoimmune disease, pediatric rheumatology, pediatric stem cell transplant

## Abstract

Juvenile systemic sclerosis (jSSc) is a rare, chronic, autoimmune disease in children/adolescents and is associated with significant morbidity, skin thickening/hardening (scleroderma), organ toxicity and sub-optimal therapeutic options. In this report, autologous stem cell transplantation is associated with clinical improvement in a 17-year-old with refractory jSSc.

## Graphical abstract

**Graphical Abstract:**
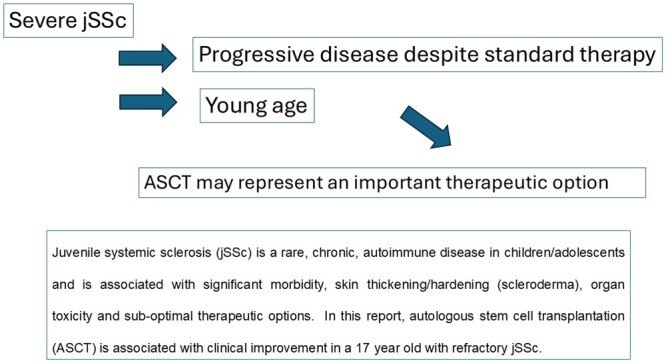

Significance statement Autologous stem cell transplantation may provide a therapeutic option for pediatric patients with treatment-resistant juvenile systemic sclerosis, offering durable clinical benefit and improved quality of life.

## Background

Juvenile systemic sclerosis (jSSc) is characterized by progressive multiorgan inflammation and fibrosis. Disease pathogenesis involves abnormal immune activation due a microvascular endothelial damage leading to excess production and deposition of collagen in various organ system leading to chronic inflammation with progressive fibrosis, which can result in severe morbidity and early mortality.^1^^,^^2^ The prevalence of jSSc is approximately 3 per 1 000 000 children, with an incidence of 0.27 per 1 000 000 children.^3^ Several organs may be affected with jSSc, including the skin with cutaneous findings such as skin tightness and thickening, which are typically assessed by the modified Rodann skin score. Other systemic manifestations can include interstitial lung disease, myocardial fibrosis, dysphagia, hypertension, and Raynaud phenomenon.^4^ Severe cases are often resistant to standard treatments, progressing to severe organ dysfunction and significant impairments in quality of life.^1^^,^^5^

Despite advances in the understanding of systemic sclerosis (SS),^2^ the management of jSSc remains challenging. Single Hub and Access point for paediatric Rheumatology in Europe has recommended initial treatment with corticosteroid treatment in addition to disease-modifying antirheumatic drugs (DMARDs) such as methotrexate in the active phase of the disease. Other immunomodulators, such as cyclophosphamide or mycophenolate mofetil, are usually used to treat lung or cardiac involvement or as second line agents in refractory cases.^6^ There is currently no standard management approach for aggressive/refractory cases; however, other biologic agents and autologous stem cell transplantation (ASCT) have been considered.^7^ ASCT has shown clinical benefit for patients with severe and progressive adult-onset systemic sclerosis in three randomized controlled trials, each demonstrating superiority of ASCT versus standard immunosuppressive therapy.^8–10^ In this population, ASCT improves skin fibrosis and stabilizes lung function while significantly prolonging progression-free and overall survival up to 5 years.^11^ Current knowledge regarding ASCT in children with jSSC is limited, with only 16 cases reported by the EBMT registry to date.^6^^,^^12^^,^^13^

## Case presentation

**Figure 1.**
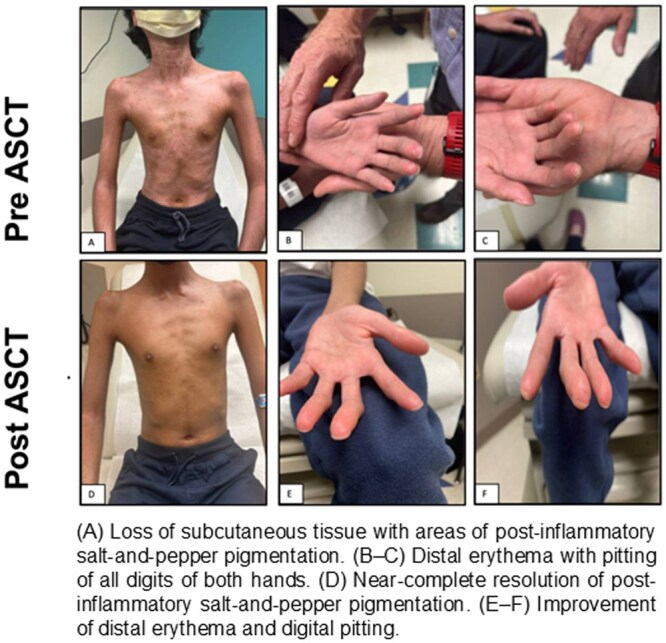
Timeline of patient’s presentation and clinical course.

A 13-year-old male presented with complaints of generalized rash, progressive bilateral knee pain, and muscle weakness. His exam was notable for shiny, tight skin over the face and extremities with hypopigmentation of the chest, neck, and back. He had Gottron’s papules over his knuckles with limited range of motion (ROM) in bilateral elbows/knees. Laboratory findings, including elevated inflammatory markers and autoimmune serology, were consistent with jSSc (score of 12 based on EULAR/ACR criteria; Figure 1).^14^ The patient initially received methotrexate and prednisone, and mycophenolate mofetil was added due to progressive weight loss, skin tightening with contractures, and difficulty breathing with restrictive lung disease. Tocilizumab was started due to further progression, and the patient had mild skin improvement. Due to persistent and progressive disease, he was referred for consideration of ASCT at 17 years of age (Figure 1).

Pre-transplant evaluation demonstrated continued restrictive lung disease, severe malnutrition, and sclerotic skin, with a modified Rodnan (mRS) score of 18 (Table 1). Despite a decline in his quality of life, the patient remained enrolled in school and exhibited sufficient organ function (including ophthalmological evaluations) to be considered suitable for transplantation (Table 1). The risks/benefits of ASCT were provided, and the family/patient consented/assented.

**Table 1.** Laboratory and work up findings pre and post ASCT.

**Studies**	Pre ASCT	1 year post ASCT
**Autoimmune work up**		
** CK**	252	61
** Anti-SCL-70**	275	>8
** Anti-SM**	8	<0.2
** Anti-DsDNA**	21	2
** Anti-RNP**	20	<0.2
** Modified Rodnan score **	18	1
**Inflammatory markers**		
** ESR**	1	35
** CRP**	<0.01	0.31
**Growth measurement**		
** BMI (kg/m²) **	13.78	14.83
** BMI percentile**	<0.01%	<0.01%
** Weight percentile**	<0.01% (34 kg)	<0.01% (37.2 kg)
** Height percentile**	0.96% (156.2 cm)	0.96% (158.4 cm)
**Organ function**		
** FEV1**	1.89 (55%)	1.92 (56%)
** Ejection fraction**	56%	60%
** GFR for cystatin C**	88 mL/min/BSA	83 mL/min/BSA
** Sleep study**	Mild OSA (AHI: 5/h)	No OSA (AHI: 0.7/h) Normal oxygenation and ventilation
** XR video swallow study with speech therapy evaluation **	Oropharyngeal dysphagia^a^	Unremarkable results^a^
**Physical assessment**		
** 6 minute walk test**	437.68 m (60% of predicted)	601.77 m (82% of predicted)
** Karnofsky performance status**	80%	80%

Normal values: CK (U/L; normal range 70-250); Anti-SCL-70 (AU/mL; normal range negative/equivocal <120); Anti-SM (AI; normal range negative <1.0); Anti- DsDNA (IU/mL, normal range negative ≤4); Anti-RNP (AU/mL; normal range negative <1.0); GFR: glomerular filtration rate for cystatin: normal >60 mL/min/BSA; ESR: erythrocyte sedimentation rate (normal range 0-13 mm/h); CRP: C-reactive protein (normal range: <0.85 mg/dL).

a With speech therapy. The patient had delayed swallow initiation with thin liquids; reduced hyolaryngeal excursion and epiglottic inversion, and trace distal esophageal retention with solid consistencies as adequate airway protection was observed with all consistencies offered. Post ASCT adequate airway protection was observed.

AHI, Apnea Hypopnea Index; DLCO, carbon monoxide diffusion capacity; FEV1, forced expiratory volume in the first second; OSA, obstructive sleep apnea; Post ASCT, patient had normal oxygenation and ventilation with some periodic limb movements.

He tolerated stem cell mobilization in one cycle with filgrastim/plerixafor without cyclophosphamide and subsequent apheresis well (CD34^+^ count: 24.28 × 10^6^ [8.62 × 10^6^/kg]).^9^ A naso-dudodenal tube was placed upon admission for ASCT to optimize nutritional status, and the patient was monitored for re-feeding syndrome with initiation of feeds. His conditioning regimen included total body irradiation (TBI) 800 cGy (shielding to allow 200 cGy to lungs and 200 cGy to kidneys), cyclophosphamide 120 mg/kg/d, and equine antithymocyte globulin (ATG) 90 mg/kg/dose with subsequent ASCT with CD34^+^ enrichment. Supportive care was provided as previously described.^9^ He achieved neutrophil engraftment on day +11 and platelet engraftment on day +12. He did not require oxygen supplementation or develop a fever/infection/major ASCT complications during his admission (other than hypertension with steroid pre-medication with ATG, which was responsive to amlodipine). He did not develop a renal crisis.

**Figure 2.**
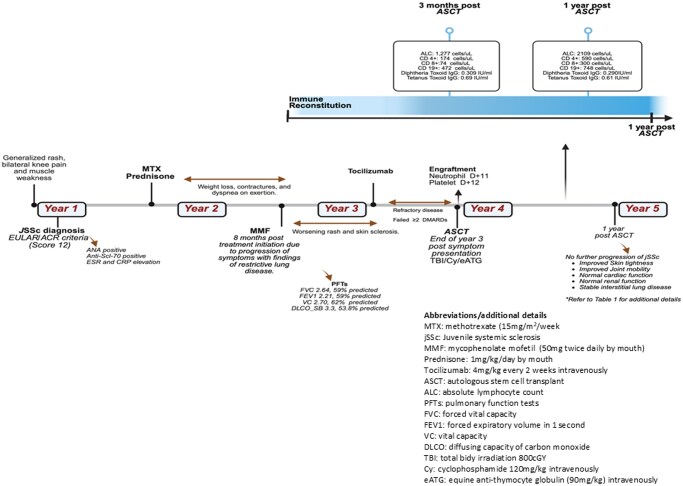
Pre and post ASCT evaluations and clinical manifestations. ASCT, autologous stem cell transplantation.

By sixteen months post-ASCT, he demonstrates improvement in mRs to 1 with significant improvement in skin tightness and joint mobility; his oral aperture increased from 29 to 36 mm (Figure 2). Moreover, he reports improvement in endurance (no school absences) and breathing, with stable interstitial lung disease. His nutritional status is stable (BMI Z score −4), and he continues physical and occupational therapy and supplemental feeds via gastrostomy tube (Table 1). He has not needed resumption of DMARDs.

## Discussion

In adults with SS, ASCT can be considered for patients with refractory progressive skin or lung disease. It has been shown to improve event-free survival (EFS) and overall survival, as well as preventing and delaying complications such as heart failure, pulmonary arterial hypertension, and the progression of lung dysfunction.^15^ Data from registries and pilot studies in Europe and the United States demonstrate significant and sustained improvement in skin fibrosis, functional ability, and stabilization of organ function following. Randomized controlled trials—American Scleroderma Stem Cell versus Immune Suppression Trial (ASSIST), Autologous Stem Cell Transplantation International Scleroderma (ASTIS), and Scleroderma: Cyclophosphamide or Transplantation (SCOT) have shown superior long-term outcomes of HSCT compared to standard cyclophosphamide therapy, with ongoing follow-up evaluating durability and late effects. While the outcomes of jSSc are better than adults, those with extensive lung and skin involvement have a 5-year mortality of 10%,^16^ therefore more studies are needed to report long-term outcomes.

In the ASSIST trial, patients received ASCT with a non-myeloablative conditioning regimen (cyclophosphamide and rabbit ATG prior to ASCT) versus six cycles of intravenous cyclophosphamide. Treatment failure, defined as disease progression without improvement, was observed in eight of the nine control patients but in none of the ten ASCT patients (*P* = .0001). After a mean follow-up of 2.6 years, all but two ASCT recipients maintained improvements in mRS and forced vital capacity, with the longest follow-up reaching 60 months.^10^ In the (ASTIS) trial, ASCT was associated with increased treatment-related mortality in the first year after treatment, likely due to the high cyclophosphamide doses used for both mobilization (4 g/m^2^ or 100 mg/kg) and transplantation (200 mg/kg) but conferred a significant long-term EFS benefit.^17^ Subsequently, the SCOT trial compared myeloablative (with fractionated total-body irradiation, cyclophosphamide, and equine ATG with pulmonary and renal shields) ASCT versus cyclophosphamide monthly × 12(9). Notably, scleroderma relapse, defined as the need for DMARD therapy, occurred in 9% of myeloablative regimen group compared to 22% in the non-myeloablative group.^9^^,^^10^^,^^17^ Transplant-related mortality in the myeloablative group was lower than previously reported (3% and 6% at 54 and 72 months post-ASCT, respectively, versus 10.7% at 1 year after non-myeloablative ASCT).^9^^,^^17^ The outcomes in 5 case reports in pediatric patients with severe refractory SS with lung involvement are encouraging, with 3 patients in remission with improvement in weight and linear growth, one in partial remission, and one patient who relapsed after 9 months.^4^^,^^18^^,^^19^ All had lung disease at inclusion, and all received the same mobilization regimen using cyclophosphamide and G-CSF with cell selection before transplantation. Varying conditioning regimens have been used including high-dose cyclophosphamide, campath, fludarabine, and antithymocyte globulin none of the patients reported to receive TBI.^18^^,^^19^ Case reports have shown that ASCT in jSSc is tolerated with significant improvement in mRS score and clinical systems, such as improvement in fatigue and daily activities.^20^

Recent guidance recommends that all types of jSSc with moderate to severe features should be considered for ASCT regardless of duration, in progressive disease or lack of improvement despite treatment with two or more DMARDS, patients with moderate to severe skin or lung disease or myopathy or cardiac involvement.^21^ In this report, the patient presented with severe skin manifestation and progressive lung disease after failing ≥2 DMARDs and thus he underwent ASCT per the SCOT trial regimen based on this center’s experience.^9^ He did not experience any clinically significant transplant related complications and showed prompt immune-reconstitution. He showed dramatic skin improvement within a year of ASCT with stabilization of interstitial lung disease (ILD), which is expected to improve with nutritional status and ongoing physical therapy. On the SCOT trial, patients showed improvement in ILD over a median of 54 months.^9^ Altogether, given the significant morbidity and impaired quality of life in patients with severe jSSc, ASCT may represent an important therapeutic option, particularly perhaps in patients who develop severe disease at a young age. Conceptually, prospective pediatric studies regarding the use of immune effector cell therapies for jSSc may represent another important area, like ASCT, that enhances an immune reset for this disease.
